# Disaster Preparedness Among Middle-Aged and Older Adults: Who is the Least Prepared?

**DOI:** 10.1093/geroni/igab046.2867

**Published:** 2021-12-17

**Authors:** Caitlin Connelly, Kathrin Boerner, Natasha Bryant, Robyn Stone

**Affiliations:** 1 University of Massachusetts Boston, Somerville, Massachusetts, United States; 2 University of Massachusetts Boston, Boston, Massachusetts, United States; 3 LeadingAge LTSS Center @UMass Boston, Washington, District of Columbia, United States; 4 LeadingAge, Washington, District of Columbia, United States

## Abstract

Adverse impacts of natural disasters are viewed as particularly concerning for older adults. Disaster preparedness is an important step towards offsetting potential harm. Research comparing different age groups with respect to their disaster preparedness has produced inconclusive evidence. Some studies found older adults more prepared than younger age groups, whereas others found them to be equally or less prepared. To shed light on this issue, we examined disaster preparedness among N = 16,409 adults age 40 and older from the American Housing Survey. Using logistic regression analyses, we compared preparedness levels of four groups – households of middle-aged adults (age 40-64), older adults (age 65-84), oldest old adults (age 85+), and mixed households comprised of both middle-aged and older adults. Findings showed that households of older adults and the oldest old had significantly higher preparedness levels compared to middle-aged and mixed households, accounting for demographics, living alone, and disability. However, the oldest old group appeared less prepared compared to the older adult group. Thus, while our findings suggest that older adults aged 65-84 may be better prepared for disasters than middle-aged adults, the oldest old group, who are likely at a higher risk of adverse impacts from natural disasters, may be less prepared than their relatively younger counterparts. Therefore, older adults should not be treated as a homogenous group when considering disaster preparedness. Rather, policies and interventions to improve disaster preparedness may benefit from focusing on specific high vulnerability groups.

